# Expression of the *GLI* family genes is associated with tumor progression in advanced lung adenocarcinoma

**DOI:** 10.1186/1477-7819-12-253

**Published:** 2014-08-08

**Authors:** Masashi Ishikawa, Makoto Sonobe, Naoto Imamura, Terumasa Sowa, Kei Shikuma, Hiroshi Date

**Affiliations:** Department of Thoracic Surgery, Faculty of Medicine, Kyoto University, 54 Shogoin-kawaharacho, Sakyo-ku, Kyoto 606-8507 Japan

**Keywords:** GLI, Hedgehog signaling, Lung adenocarcinoma, Advanced stage, Expression, Prognosis

## Abstract

**Background:**

The hedgehog (Hh) signaling pathway is aberrantly activated in various cancers. Expression of the GLI family of genes, which encode for transcriptional factors of the Hh pathway, has not been fully assessed in clinical samples of advanced lung adenocarcinoma. In this study, we retrospectively evaluated the expression of the GLI family of genes in advanced stage lung adenocarcinoma samples and determined their relation to patient survival.

**Methods:**

The levels of *GLI1*, *GLI2*, and *GLI3* mRNA expression were measured by quantitative real-time polymerase chain reaction in surgically obtained tissue samples from stage II-IV lung adenocarcinoma patients (n = 102). Pairwise comparisons between all three *GLI* mRNA expression were performed, and after dichotomizing the patients into low and high expression groups according to each *GLI* mRNA expression level, survival curves were calculated and multivariate analyses were conducted.

**Results:**

Significant positive correlation was found between *GLI1* and *GLI3* mRNA expression (*P* <0.001). Tumors with higher expression (upper 15%) of *GLI1* or *GLI3* mRNA were associated with poor survival in stage II-IV (5-year overall survival rates: *GLI1* mRNA low, 41.7% *vs.* high, 20.0%, *P* = 0.0074; *GLI3* mRNA low, 43.1% *vs.* high, 13.3%, *P* = 0.0062) and stage III-IV (5-year overall survival rates: *GLI1* mRNA low, 34.0% *vs.* high, 0%, *P* = 0.0012; *GLI3* mRNA low, 33.4% *vs.* high, 7.7%, *P* = 0.057) lung adenocarcinoma patients. *GLI2* mRNA expression did not appear to have great clinical significance. Multivariate analysis revealed higher *GLI1* mRNA expression as an independent factor for unfavorable patient survival (*P* = 0.0030, hazard ratio = 3.1, 95% confidence interval = 1.5-6.2), as well as tumor differentiation and stage.

**Conclusions:**

Expression of *GLI1* and *GLI3* mRNA was strongly correlated, and their overexpression, especially that of *GLI1*, was found to be predictive of aggressive tumor behavior. This study indicates that the Hh pathway may be a key oncogenic signaling network in tumor pathogenesis and, thus, a potential therapeutic target in advanced lung adenocarcinoma.

## Background

Non-small cell lung cancer (NSCLC) has been the leading cause of cancer-related deaths world-wide, and adenocarcinoma accounts for the majority of all NSCLC cases [1, 2]. Although any possibility of a cure rests almost entirely with surgical resection with or without chemo- or radiotherapy, postoperative recurrence rate is still high and survival rate remains low compared with other types of cancers. In particular, advanced NSCLC can be exceptionally lethal even with aggressive anticancer therapies, despite the dramatic treatment shift of some targeted therapeutic agents for lung adenocarcinoma [3, 4]. Developing and deploying new treatment approaches are vital to improve patient prognoses.

The hedgehog (Hh) signaling pathway, which consists of three distinct homologues (sonic, indian, and desert Hh) is constitutively activated in a variety of human tumor entities [5]. Normally, Hh signaling plays an important role during vertebral embryonic development and tissue maintenance [6]. The Hh proteins are secreted molecules, and their receptor, Patched, usually functions as a downstream inhibitor in the absence of Hh. Hh signaling is activated through binding to the Patched receptor, which unleashes the transmembrane protein, Smoothened, and ultimately upregulates GLI [5]. GLI proteins are zinc finger transcription factors which exert their functions by nuclear localization and promoting target gene transcription, and in humans, at least three distinct *GLI* genes, *GLI1*, *GLI2*, and *GLI3*, have been identified [7]. As the GLI family of proteins are the final mediators of Hh signaling, their expression, especially that of GLI1, is deemed to be the best reflection of the net effect of Hh activation, thus, survival studies on GLI may indicate the potential involvement of Hh in tumor pathogenesis [8].

In addition to the tumorigenesis based on genetic alterations of the Hh pathway predominantly seen in basal cell carcinoma and medulloblastoma, extensive involvement of ligand-dependent Hh activation has been suggested in various cancer entities [9, 10]. With regard to lung cancer, while the involvement of Hh signaling in small cell lung cancer has been shown to be evident [11–13], it remains poorly understood in lung adenocarcinoma, especially in the advanced stages.

In this study, we evaluated the expression levels of the three *GLI* family genes (*GLI1*, *GLI2*, and *GLI3*) in surgical samples from advanced lung adenocarcinoma patients (stage II-IV), and revealed the possible involvement of the Hh signaling pathway in tumor advancement by analysis for correlations between GLI expression and patient prognosis. As we found considerable impact of *GLI1* and *GLI3* gene expression on patient survival, this study demonstrates that inhibition of Hh signaling may be a promising drug target for the treatment of advanced lung adenocarcinoma.

## Methods

### Patients, tissue samples, and tumor information

Tissue samples were obtained from patients who were diagnosed as stage II-IV lung adenocarcinoma after surgical resection at the Kyoto University Hospital between July 2001 and December 2007. Continuous surgical cases with sufficient tissue material for evaluations were included in this study (n = 102). All of these tumors were histologically confirmed as lung adenocarcinoma, and their tumor staging and degree of differentiation were all assessed by board-certified pathologists in the Department of Pathology of the Kyoto University Hospital. Tumor stage was determined by the latest tumor-node-metastasis classification system [14]. Histological type and grade of cell differentiation were determined according to the WHO classification system [15]. Their demographics and baseline characteristics are presented in Table 1. Pre- or postoperative chemotherapy was mainly composed of orally administered tegafur-uracil and/or conventional intravenous chemotherapeutic agents (carboplatin, paclitaxel, docetaxel, gemcitabine, vinorelbine) for NSCLC. Only one patient (stage II) received radiotherapy prior to surgery (concurrently with chemotherapy), and postoperative radiotherapy (mediastinum or chest wall) was performed in four patients (stage III). Complete tumor resection was achieved in 77 patients (75.5%), whereas in 25 patients (24.5%) macro- or microscopic residual tumors were suspected after surgery (for example, pleural dissemination, malignant pleural effusion, positive stumps, or distant metastasis prior to surgery). Informed consent for participation in this study was obtained from all patients prior to their surgeries, and this study was reviewed and approved by the Ethics Committee of the Graduate School and Faculty of Medicine at the Kyoto University.

**Table 1:** **Characteristics of the patients included in the study**

		Numbers (n)	Percent (%)
Age (years)			
	Mean ± SD (range)	64.8	±9.8 (31-81)
Gender			
	Male	68	66.7
	Female	34	33.3
Cigarette smoking			
	Never smoked	34	33.3
	Ex-smoker	20	19.6
	Current smoker	48	47.1
	Pack-year/Mean ± SD (range)	36.6	±36.8 (0-180)
Tumor differentiation			
	Well	16	15.7
	Moderate	53	52.0
	Poor	32	31.4
	Unknown	1	
Tumor stage			
	II	31	30.4
	III	60	58.8
	IV	11	10.8
Operation method			
	Pneumonectomy	1	1.0
	Bilobectomy	1	1.0
	Lobectomy	83	81.4
	Segmentectomy	9	8.8
	Partial resection	8	7.8
Complete resection			
	Yes	77	75.5
	No	25	24.5
Preoperative chemotherapy			
	Yes	9	8.8
	No	93	91.2
Postoperative chemotherapy			
	Yes	74	72.5
	No	28	27.5
Radiotherapy to thorax (chest wall or mediastinum)^a^			
	Yes (Pre/Post)	5 (1/4)	4.9
	No	97	95.1
*EGFR* gene mutation status			
	Wild-type	46	45.1
	Mutation (+)	33	32.4
	Unknown	23	22.5
Recurrence^b^			
	Yes	82	80.4
	No	20	19.6
Prognosis			
	Alive	33	34.4
	Dead	63	65.6

^a^Not including palliative radiotherapy.

^b^Including residual tumor after surgery.

SD: standard deviation.

### Preparation of tissue mRNA

For sample collection, tumor tissue samples were dissected immediately after surgical resection and soaked in RNAlater TissueProtect Tubes (Qiagen, Tokyo, Japan) for more than 48 h before storage at -80°C until use. Total RNA was isolated from tissue samples using RNeasy Plus Mini Kit (Qiagen), and reverse transcription of total RNA was conducted using the Ready-To-Go You-Prime First-Strand Beads (Amersham Biosciences, Uppsala, Sweden) to obtain cDNA.

### Quantification of *GLI*mRNA

To quantify *GLI* mRNA expression levels of each sample, quantitative real-time PCR was performed using the LightCycler thermal cycler system (Roche Diagnostics Japan, Tokyo, Japan). The PCR primers used for the quantitative amplification of *GLI1* mRNA were forward: 5′- CTCCCGAAGGACAGGTATGTAAC -3′ and reverse: 5′- CCCTACTCTTTAGGCACTAGAGTTG -3′, those of *GLI2* mRNA were forward: 5′- AGAAGCAGCGCAATGACGTG -3′ and reverse: 5′- GTCATCCAGTGCCGTCAGGT -3′, and those of *GLI3* mRNA were forward: 5′- AAACCCCAATCATGGACTCAAC -3′ and reverse: 5′- TACGTGCTCCATCCATTTGGT-3′. The primers for *glyceraldehyde-3-phosphate dehydrogenase* (*GAPDH*) mRNA, used as an internal control, were forward: 5′-ACAACAGCCTCAAGATCATCAG-3′ and reverse: 5′-TCTTCTGGGTGGCAGTGATG-3′. After a 20-μL reaction mixture containing 0.5 μM forward/reverse primers and 0.03 μg cDNA in QuantiTect SYBR Green PCR Master Mix (Qiagen) was prepared, PCR amplification was initiated by preincubation for 15 min at 95°C for initial activation, followed by 40 cycles of the following protocol: denaturation at 94°C for 15 s, annealing at 59°C for 15 s, and elongation at 72°C for 15 s with detection of fluorescence products. The quantitative data were analyzed with LightCycler analysis software version 5.03 (Roche Diagnostics Japan). The expression levels of *GLI* genes were calculated as the ratio of *GLI* mRNA value to *GAPDH* mRNA value, and are expressed as median and mean ± standard deviation.

### Statistical analysis

Relationships between each pair of mRNA expression were estimated by calculating the Spearman’s rank correlation coefficient (r_s_). Pairwise comparisons of postoperative survival of each GLI low and high groups were compared using the Mann-Whitney U test. Survival curves were evaluated by the Kaplan-Meier method, and cumulative survival data were compared using the log-rank test. Multivariate analysis of prognostic factors was performed by the Cox proportional hazard model. Differences were considered significant when *P* <0.05. All statistical analyses were performed using StatMate IV software version 4.01 (ATMS, Tokyo, Japan) and JMP software version 8 (SAS Institute Japan, Tokyo, Japan).

## Results

### *GLI1*, *GLI2*, and *GLI3*mRNA were expressed in almost all advanced lung adenocarcinoma tissues

**Figure 1:**
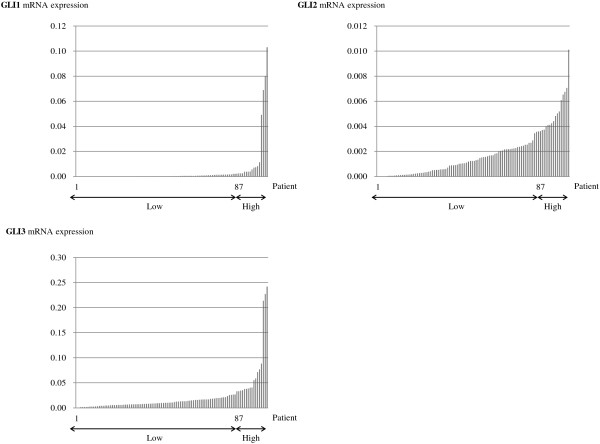
***GLI*** **mRNA expression in stage II-IV lung adenocarcinoma patients.** The levels of *GLI1*, *GLI2*, and *GLI3* mRNA expression were measured by quantitative real-time polymerase chain reaction in stage II-IV lung adenocarcinoma samples. The three *GLI* mRNA were detected in all 102 samples, except three patients who were negative for GLI2. Wide variations were observed in *GLI1* and *GLI3* mRNA levels. The top 15% of patients with the highest GLI expression were defined as the higher expression group in each category. Each mRNA value was expressed as a ratio to *GAPDH* mRNA (internal control).

*GLI1* and *GLI3* mRNA expression was detected in all 102 samples, whereas *GLI2* mRNA was detected in 99 samples. The level of *GLI1* mRNA (normalized and expressed as a ratio to *GAPDH* mRNA) ranged from 2.35 × 10^-6^ to 1.03 × 10^-1^ (median, 3.03 × 10^-4^; mean ± standard deviation, 3.97 × 10^-3^ ± 1.51 × 10^-2^); *GLI2* mRNA level was 0 to 1.01 × 10^-2^ (1.25 × 10^-3^, 1.78 × 10^-3^ ± 1.86 × 10^-3^); and *GLI3* mRNA level was 7.68 × 10^-5^ to 2.42 × 10^-1^ (1.07 × 10^-2^, 2.23 × 10^-2^ ± 3.93 × 10^-2^). The three *GLI* mRNA expression levels in all tissue samples are shown in Figure 1. Wide variations were observed in *GLI1* and *GLI3* mRNA levels in the primary tumor cohort, and based on these, we defined the upper 15% of patients as high *GLI* mRNA expression for each *GLI* gene (cutoff line; *GLI1* mRNA: 2.49 × 10^-3^, *GLI2* mRNA: 3.71 × 10^-3^, *GLI3* mRNA: 3.46 × 10^-2^).

### *GLI1*and *GLI3*mRNA expression levels were remarkably correlated

**Figure 2:**
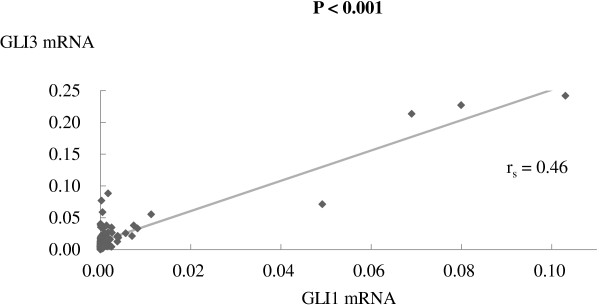
**Relationship between** ***GLI1*** **and** ***GLI3*** **mRNA expression in stage II-IV lung adenocarcinoma patients.** Pairwise comparisons of each *GLI* mRNA expression level showed that there was a strong positive correlation between *GLI1* and *GLI3* mRNA expression (r_s_ = 0.46, *P* <0.001). Statistical significance was estimated by calculating the Spearman’s rank correlation coefficient (r_s_). Each mRNA value was expressed as a ratio to *GAPDH* mRNA (internal control). Bold value: statistically significant.

By comparing *GLI1*, *GLI2*, and *GLI3* mRNA expression levels, strong positive correlation was observed between *GLI1* and *GLI3* mRNA expression (r_s_ = 0.46, *P* <0.001) (Figure 2), whereas no such relations were found between *GLI2* and *GLI1* or *GLI3* mRNA expression levels. Even with exclusion of the four observed outliers (n = 98), strong positive correlation was also found between *GLI1* and *GLI3* mRNA expression (r_s_ = 0.40, *P* <0.001).

### *GLI1*and *GLI3*mRNA expression reflected postoperative patient survival

**Figure 3:**
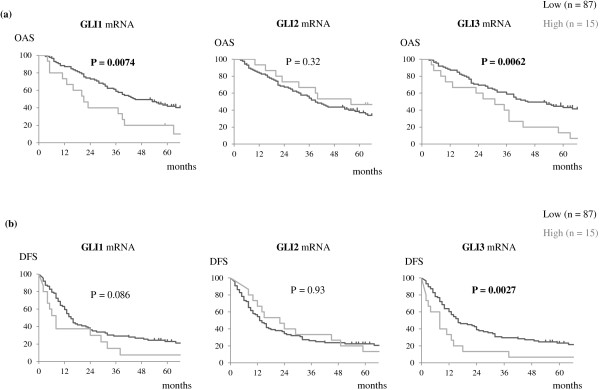
**Survival curves according to** ***GLI*** **mRNA expression in stage II-IV lung adenocarcinoma patients.** Kaplan-Meier analysis was performed on the basis of *GLI* mRNA expression in all cohorts (n = 102). *P* values were estimated by log-rank test. Tumors with higher expression (upper 15%) of *GLI1* or *GLI3* mRNA were associated with poor survival in patients with stage II-IV lung adenocarcinoma. No statistical significance was observed for *GLI2* mRNA expression. **(a)** Overall survival; **(b)** Disease-free survival. Ticks: censored cases; Bold values: statistically significant.

There were statistically significant differences in postoperative survival between *GLI1*/*GLI3* low and high mRNA expression groups (*GLI1* mRNA: *P* <0.001; *GLI3* mRNA: *P* <0.001). Survival curves for each high/low *GLI* mRNA expression groups are presented in Figure 3. Five-year overall survival (OAS) rates in the *GLI1* mRNA low and high groups were 41.7% and 20.0%, and those in the *GLI3* mRNA low and high groups were 43.1% and 13.3%, respectively. Higher *GLI1* and *GLI3* mRNA expressing tumors in advanced lung adenocarcinoma patients were correlated with significantly worse overall survival rates than lower expression groups (*GLI1* mRNA: *P* = 0.0074, hazard ratio (HR) = 2.2 (95% confidence interval: 1.4-6.9); *GLI3* mRNA: *P* = 0.0062, HR = 2.2 (1.4-6.6)). Similarly, 5-year disease-free survival (DFS) rates in the *GLI1* mRNA low and high groups were 22.9% and 7.5% (*P* = 0.086; HR = 1.7 (0.91-3.9)), and those in the *GLI3* mRNA low and high groups were 23.2% and 6.7% (*P* = 0.0027; HR = 2.3 (1.5-7.1)), respectively. With regard to *GLI2* mRNA expression, no statistical significance was observed between high and low expression groups (OAS: *P* = 0.32; DFS: *P* = 0.93).

### Higher *GLI1*mRNA expression was correlated with significantly worse prognosis especially in stage III-IV lung adenocarcinoma patients

**Figure 4:**
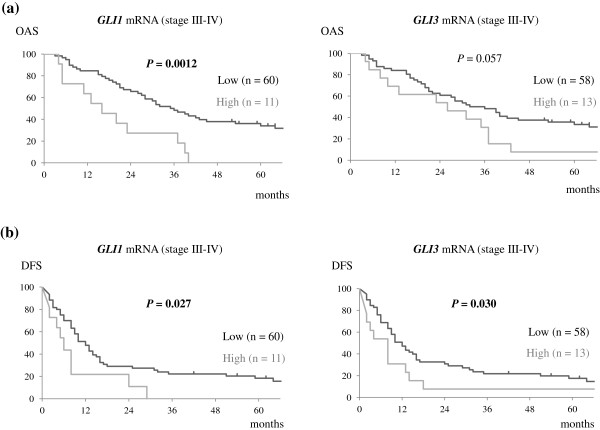
**Survival curves according to** ***GLI1*** **and** ***GLI3*** **mRNA expression in stage III-IV lung adenocarcinoma patients.** Kaplan-Meier analysis was performed on the basis of *GLI1* and *GLI3* mRNA expression in patients with stage III-IV lung adenocarcinoma. *P* values were estimated by log-rank test. Higher *GLI1* and *GLI3* mRNA expression also correlated with unfavorable patient survival. **(a)** Overall survival; **(b)** Disease-free survival. Ticks: censored cases; Bold values: statistically significant.

Subset analysis of patients with stage III-IV lung adenocarcinoma (n = 71) showed significantly worse clinical outcomes in both the higher *GLI1* and *GLI3* mRNA expression groups. In this cohort, 5-year OAS rates in the *GLI1* mRNA low and high groups were 34.0% and 0%, respectively (*P* = 0.0012, HR = 2.8 (1.9-13.5)), and 5-year DFS rates in these groups were 18.3% and 0%, respectively (*P* = 0.027, HR = 2.1 (1.1-7.2)). Although the difference in OAS between the *GLI3* mRNA expression groups was marginal (low, 33.4% *vs.* high, 7.7%, *P* = 0.057), 5-year DFS rates were statistically different between *GLI3* mRNA low and high groups (low, 17.6% *vs.* high, 7.7%, *P* = 0.030, HR = 1.9 (1.1-5.3)). Survival curves are presented in Figure 4.

### Higher *GLI1*mRNA expression was an independent prognostic factor in advanced lung adenocarcinoma

The results of multivariate analyses for each clinicopathological parameter are presented in Table 2. Higher *GLI1* mRNA expression was demonstrated to be an independent prognostic factor for poor patient prognosis (*P* = 0.0030, HR = 3.1 (1.5-6.2)) as well as tumor differentiation (well *vs.* poor) and tumor stage. No correlations were found for *GLI2* and *GLI3* mRNA expression with other clinicopathological features.

**Table 2:** **Multivariate analysis of** ***GLI1*** **mRNA expression and other clinicopathological variables for patient survival**

Variables		Multivariate analysis		
		Hazard ratio	95% CI	***P-***value ^a^
*GLI1* mRNA expression				
	Low	1		**0.0030**
	High	3.1	1.5-6.2	
Age (years)				
	≤65	1		0.23
	>65	1.3	0.83-2.2	
Gender				
	Male	1		0.86
	Female	0.92	0.37-2.2	
Cigarette smoking				
	Never smoked	1		0.69
	Smoker (ex or current)	1.2	0.48-3.1	
Tumor differentiation				
	Well	1		
	Moderately	1.7	0.83-3.6	0.16
	Poorly	2.5	1.1-5.8	**0.030**
Tumor stage				
	II	1		
	III	2.5	1.5-4.5	**<0.001**
	IV	4.2	1.7-9.8	**0.0027**
*EGFR* gene mutation status				
	Wild-type	1		
	Mutation (+)	1.1	0.58-2.0	0.77
Pre/postoperative chemo- and/or radiotherapy				
	No	1		
	Yes	0.67	0.33-1.4	0.29

*Cox’s proportional hazard model.

CI: confidence interval.

Bold values are statistically significant.

## Discussion

Previous reports on Hh molecules have already provided us with convincing information on its etiologic contribution to NSCLC [16]. A large percentage of primary NSCLC cell lines have been shown to express Hh target genes including GLI1, indicating constitutive activation of the Hh pathway in NSCLC cell lines [17]. It was recently shown that disrupting the Hh pathway inhibited NSCLC proliferation *in vitro* and abrogated tumor growth *in vivo* [18]. In clinical samples, immunohistochemical analysis of tissue microarray have revealed significant correlation among the Hh molecules in early-stage NSCLC and of lymph node metastasis with nuclear GLI1 immunolocalization in lung adenocarcinoma [19, 20]. However, the impact of Hh signaling on patient survival has not been previously reported in NSCLC.

In this study, expression of the three *GLI* genes was demonstrated in almost all cases in this cohort, although their expression levels varied widely. Interestingly, some lung adenocarcinoma samples showed relatively high *GLI1* and *GLI3* mRNA expression, and their expression patterns were considerably correlated. Although GLI1 is considered to be the major effector in Hh signaling, the role of GLI3 is still controversial. Whereas GLI3 acts mainly as a repressor in normal Hh signaling [21], some reports have suggested its activator capacity in oncogenesis [22, 23]. The present study also provides evidence of the capacity of GLI3 to activate Hh signaling in concert with GLI1 in advanced lung adenocarcinoma. With regard to GLI2, very little is characterized about its expression in NSCLC, and only correlation between nuclear GLI2 with sonic Hh expression in lung squamous cell carcinoma has been reported [19]. From our research, no relation between *GLI2* mRNA expression and *GLI1* mRNA level, *GLI3* mRNA level, or patient survival was detected, thus, the role of GLI2 in the pathogenesis of lung adenocarcinoma is still unclear.

While GLI1 is mainly regulated at the transcriptional level, GLI2 is considered as a latent transcriptional regulator that can be activated by Hh signaling. Modification of the N-terminus of GLI2 is a critical step in the regulation of the transcriptional activity of the protein [8]. This as well as the difference in cancer types may explain the discrepancies in GLI2 expression and patient outcomes between our results and previous ones [24]. Our research is based on the genetic expression of GLI, whereas for GLI2, it may be necessary to assess its functional protein level instead.

The mechanisms by which Hh signaling is involved in cancer pathogenesis are not uniform, and there are several different models of Hh signal transduction. Besides the most evident pattern of ligand-independent mutational Hh activation seen in basal cell carcinoma and medulloblastoma, ligand-dependent autocrine Hh activation in tumor cells has also been shown to be common in many cancers including NSCLC. In addition, ligand-dependent paracrine Hh signaling that involves the tumor microenvironment can lead to tumor development [25]. Crosstalk with other developmental cascades such as Wnt or Notch is also frequently observed [26]. This diversity of Hh signaling in cancer development in part explains the differences across cancer types [27, 28].

Previous reports on expression profiles of Hh-related molecules in NSCLC specimens had focused mainly on the estimation of protein levels by immunohistochemistry [19, 20], and their gene expression has not been thoroughly investigated. This study is unique in that thorough investigation was performed for the gene expression of all three GLI family members, which enabled us to directly compare their levels in clinical samples and to reveal the unexpected correlation between *GLI1* and *GLI3* mRNA expression. Interestingly, some GLI1/GLI3 highly expressing tumors (in this cohort, defined as the upper 15th percentile of patients for each mRNA expression) showed significantly poorer patient prognosis in advanced lung adenocarcinoma. Although a statistically significant negative correlation between survival and GLI1 expression has been shown in breast [29], esophageal [30], colorectal [31], and bladder cancers [32], this is the first study to demonstrate a clinical impact of GLI1 on patient survival in lung adenocarcinoma. Our speculation was further supported by the multivariate analysis, which showed that *GLI1* mRNA expression is an indicator for unfavorable prognosis. Although the independency of Hh molecule expression on patient survival has been previously shown in bladder and prostate cancers [32, 33], we are the first to reveal that of GLI1 in lung adenocarcinoma, which is indicative of the involvement of Hh signaling in a subset of NSCLC. Investigation on GLI3 expression in tumor samples has been rare, and the possible clinical impact of GLI3 expression raised by our study needs further corroboration by additional research studies.

Irrespective of these novel observations, some limitations should be addressed in the present study. First, the validity of the cutoff of mRNA values was not ensured. Unfortunately, for our data, dichotomizing patients by either median or mean value of mRNA expression provided little information on survival difference, thus, we defined the upper 15% expression level as the cutoff. Further, patient backgrounds were diverse. Due to the retrospective nature of analysis of these advanced stage cancer cases, pre- or postoperative therapies were not standardized and the completeness of resection was not fully warranted (for example, in stage IV patients, operations were mainly performed for salvage or diagnosis). We believe that these limitations can be overcome by expanding patient numbers and/or careful selection of patients with similar clinical backgrounds. Additionally, analyzing the expression profiles of GLI proteins as well as that of other Hh molecules in this cohort would further facilitate the deepening of our understanding.

With the advent of small molecule Hh inhibitors, such as vismodegib [34, 35], recent interest has focused on the clinical application of Hh inhibitors in various types of tumors harboring aberrantly activated Hh signaling [25, 36]. Based on our results, Hh inhibition may be a good candidate treatment for advanced lung adenocarcinoma. Because a similar study on stage I-II lung adenocarcinoma did not show any significance on patient survival [19], this signaling pathway may be involved more in tumor progression rather than tumorigenesis, and disrupting Hh may be more efficacious in the advanced stages of lung adenocarcinoma. Further studies including analysis of the expression of other Hh molecules or on other histological types of NSCLC are necessary to provide extended support of our findings.

## Conclusions

We found that *GLI1*, *GLI2*, and *GLI3* mRNA were expressed in almost all advanced stage lung adenocarcinoma tissue samples, and strong correlation was observed between *GLI1* and *GLI3* mRNA expression. While *GLI2* mRNA expression level was of little clinical significance, a subset of patients showing relatively high *GLI1*/*GLI3* expression showed poorer patient survival. In particular, higher *GLI1* mRNA expression was an independent prognostic factor for unfavorable prognosis. This is the first report to describe the clinical involvement of the GLI family of genes in advanced lung adenocarcinoma, and these results suggest that Hh signal inhibition is a good candidate therapeutic target for advanced stage lung adenocarcinoma, which provide an impetus for further investigations on the Hh signaling in NSCLC.

## Abbreviations

CI: Confidence interval
DFS: Disease-free survival
GAPDH: Glyceraldehyde-3-phosphate dehydrogenase
Hh: Hedgehog
HR: Hazard ratio
NSCLC: Non-small cell lung cancer
OAS: Overall survival
r_s_: Spearman’s rank correlation coefficient
SD: Standard deviation.
